# Minimally Disruptive Medicine: A Pragmatically Comprehensive Model for Delivering Care to Patients with Multiple Chronic Conditions

**DOI:** 10.3390/healthcare3010050

**Published:** 2015-01-29

**Authors:** Aaron L. Leppin, Victor M. Montori, Michael R. Gionfriddo

**Affiliations:** 1 Knowledge and Evaluation Research Unit, Mayo Clinic, 200 First Street SW, Rochester, MN 55905, USA; E-Mails: montori.victor@mayo.edu (V.M.M.); gionfriddo.michael@mayo.edu (M.R.G.); 2 Department of Health Sciences Research, Mayo Clinic, 200 First Street SW, Rochester, MN 55905, USA; 3 Mayo Graduate School, Mayo Clinic, 200 First Street SW, Rochester, MN 55905, USA

**Keywords:** minimally disruptive medicine, chronic disease, multiple chronic conditions, healthcare delivery, care delivery models, complexity

## Abstract

An increasing proportion of healthcare resources in the United States are directed toward an expanding group of complex and multimorbid patients. Federal stakeholders have called for new models of care to meet the needs of these patients. Minimally Disruptive Medicine (MDM) is a theory-based, patient-centered, and context-sensitive approach to care that focuses on achieving patient goals for life and health while imposing the smallest possible treatment burden on patients’ lives. The MDM Care Model is designed to be pragmatically comprehensive, meaning that it aims to address any and all factors that impact the implementation and effectiveness of care for patients with multiple chronic conditions. It comprises core activities that map to an underlying and testable theoretical framework. This encourages refinement and future study. Here, we present the conceptual rationale for and a practical approach to minimally disruptive care for patients with multiple chronic conditions. We introduce some of the specific tools and strategies that can be used to identify the right care for these patients and to put it into practice.

## 1. Introduction

The healthcare resources of the United States are vast, but the value of the care that they generate is a topic of debate and concern [1]. An increasing proportion of healthcare resources are directed toward an expanding group of complex and multimorbid patients [2]. Improving the value of care for these patients (e.g., improving the quality and experience of care and reducing its cost) has become a national priority [3].

Minimally disruptive medicine (MDM) is a patient-centered approach to care that focuses on achieving patient goals for life and health [4] while imposing the smallest possible treatment burden on patients’ lives. It is particularly appropriate for patients who are at risk of being (or who already are) overwhelmed by the demands of life, illness, and health care. Such patients include the expanding group of vulnerable individuals with multiple chronic conditions [2].

In formulating treatment programs, MDM seeks to rightsize and redirect care strategies to fit patient context and be minimally disruptive and maximally supportive. In the setting of multimorbidity, this often requires the adjustment of protocols and practice guidelines to fit evidence-based patient needs, informed patient wants, and complicated patient circumstances. In this paper, we present the theoretical basis for the MDM Care Model and outline the practical strategies and tools that can be used to fit health care to the lives and goals of patients with multiple chronic conditions (see Box 1).

Box 1 **A fictitious patient, Debbie.**

Debbie is a 55 year-old patient with type 2 diabetes, mild depression, and a history of breast cancer for which she was surgically treated 3 years ago. She is divorced and lives in a small apartment with her teenage son and her cat. Debbie works as a teller at a bank where she has been stably employed for the past 2 years. She enjoys writing, reading, and art of all kinds. Debbie is overweight but has been in generally good health. She tries to go walking around her neighborhood for exercise. She takes oral medications for her diabetes, and an aromatase inhibitor for her history of breast cancer.

Debbie presents to her primary care physician for follow-up of a recent emergency room visit for a sprained ankle. Her blood glucose was elevated in the ER and an HbA1c drawn in the clinic is 8.0%. Debbie’s clinician takes a standard history and physical and suggests she be transitioned to insulin therapy. An appointment is scheduled with a diabetes educator for training on diabetes self-management and insulin use.

## 2. Minimally Disruptive Medicine: The Rationale

MDM manifests as an expression of care for and about the whole person. It recognizes the capabilities that complex patients and caregivers have to attend to healthcare demands and honors the implications of this attention on the pursuit of those goals that are most meaningful to patients: e.g., to be someone, to do something, to live some way. In MDM, professionals work with patients and caregivers to design care that advances patient goals with the smallest possible healthcare footprint on their lives: this is the primary objective of MDM.

To achieve this patient-centered objective, the MDM Care Model makes effective use of 2 deceptively simple strategies. These are: (1) to identify the right care and, (2) to make the right care happen. Although the rationale for these strategies is obvious, neither is effectively pursued in the routine delivery of health care [5]. It is thus a key point of the MDM Care model that the intention of its operation—to deliver the best care possible to patients—is not novel. What is new are its theory-based conceptualization of what right care is in the setting of multimorbidity and its prescription for how to make it happen. These key distinctions help to orient a comprehensive and testable model for practical and effective healthcare delivery for patients with multiple chronic conditions.

## 3. Strategy 1: Identify the Right Care

### 3.1. Acknowledge the Work

Prerequisite and unique to the understanding or practice of MDM is a patient-centered acknowledgement of the “hard work” that patienthood requires [4,6]. In the setting of chronic conditions, this work often goes unrecognized or underappreciated [7], although it is estimated to demand, every day, 2 hours of a patient’s attention and effort [8]. For example, patients with chronic conditions are often expected to (a) make sense of their health conditions, tests, and treatments; (b) enroll support, plan to attend healthcare visits and enact self-care activities; (c) operationalize the work of attending visits and self-managing their care; and (d) monitor, appraise, and evaluate the worth of the work they are doing [9]. May and colleagues have described the work involved in acquiring new routines and the ways in which patients are able (or unable) to enact prescribed work into their lives [10,11]. By “normalizing” the work of being a patient into everyday life and routine, patients can exhibit resilience, institute treatments with high fidelity, and achieve therapeutic success. Key components of an MDM approach to care are an awareness of the work that multimorbid patients and their caregivers must do, an understanding of the need for this work to be embedded in daily routines (and of how life circumstances can interfere with this process), and the implementation of strategies that will make care more workable to the life and context of these individuals.

### 3.2. Acknowledge the Capacity

Every structure or system (including patients with multiple chronic conditions and their caregivers) has a maximum performable workload that is determined by its unique capacity (e.g., a physician can only treat so many patients, a researcher can only manage so many studies, a student can only handle so many courses). Capacity itself can be thought of as the sum total of resources and abilities that a patient can draw on to access care, use care, and enact self-care. It comprises physical, mental, social, financial, personal, and environmental domains and is dynamic, changing with one’s disease and life trajectory: e.g., a heart failure exacerbation reduces physical capacity, the loss of a job impacts financial capacity, complicated family situations tax a patient’s mental bandwidth. Depending on an individual’s unique resilience—a component of personal capacity—the adequacy of total capacity may be judged and experienced in unpredictable ways [12]. When capacity is insufficient to carry out the work of life and health care, patients with multiple chronic conditions will not thrive. Thus, practitioners of MDM must take intentional steps to understand all patient-experienced deficits in capacity.

### 3.3. Acknowledge the Complexity

Chronic care delivery, even in the most advanced healthcare settings, is largely organized to achieve guideline-based clinical targets for single conditions (e.g., HbA1c < 7% in patients with type 2 diabetes). This approach is insensitive to patient context [13,14,15] and operates under the implicit assumption that single care strategies are universally applicable to all patients that meet a certain criterion (and that multiple, individual strategies apply when multiple criteria are met—as is typically the case in patients with multiple chronic conditions). In reality, however, every patient with a chronic disease exists within a unique biopsychosocial context, and an increasing number have several coexisting chronic health conditions. Because these factors are practically relevant and known to affect outcomes (often interacting in large and unpredictable ways) [13,16,17], the MDM Care Model is designed to account for them.

Indeed, MDM works from the perspective that patients with multiple chronic conditions (and their corresponding care delivery teams) exist as “complex adaptive systems.” Such systems are characterized, according to mathematical theory, by a large number of interactive components—such as disease and contextual variables—and by their ability to exhibit emergent properties, such as a global clinical picture or a unique clinical syndrome [18,19,20,21,22]. Mapping patient context to pre-existing mathematical models of complexity helps to orient the study and practice of MDM. Indeed, when the demands of health care and life exceed the capacity of complex patients with multiple chronic conditions, the result is a clinical syndrome (*i.e.*, an emergent property) that we describe as a workload-capacity imbalance. Practitioners of MDM are trained to prevent, diagnose, and treat this syndrome as a manifestation of patient-centered care and to improve care effectiveness.

### 3.4. Integrate the Inputs

The ways in which workload, capacity, and complexity relate to affect patient outcomes are outlined in the Cumulative Complexity Model [23], the conceptual framework that defines the workload-capacity balance and practically orients MDM-based care.

**Figure 1:**
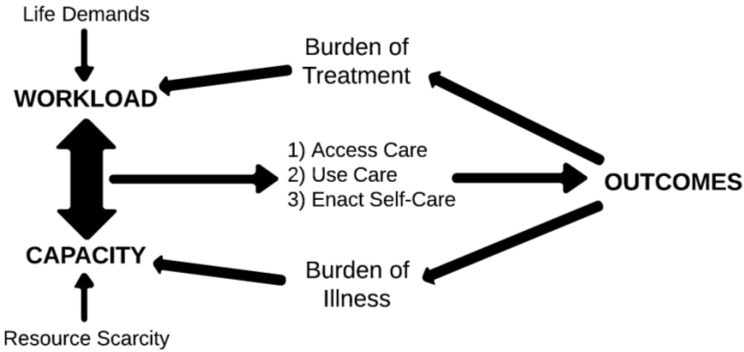
The cumulative complexity model.

As indicated by Figure 1, when workload exceeds capacity, patients feel the effects of treatment burden and this can lead to de-prioritization of various aspects of care and ultimately result in poor fidelity to treatment programs and to treatment failures. This balance has been explicitly used to explain why some patients get readmitted to the hospital [24].

The optimal method of moving a complex system through time is one that is flexible, adaptive, and coherent [25]. As such, the MDM Care Model, in its efforts to shift the workload-capacity balance in favor of patients and the achievement of their goals, attempts to flexibly integrate a number of patient contextual inputs and generate a coherent and individualized response.

MDM promotes and facilitates the “practice” of medicine; an effort that is widely regarded as important and relevant but, ironically, often not incentivized or strategically leveraged [26,27]. Indeed, for chronic disease care to be truly effective for patients with multiple chronic conditions, it must be holistic, sensitive to context, and capable of accounting for and addressing the complex ways in which relevant factors exist and interact: this requires wisdom and empathy—distinctly human characteristics—at multiple levels.

Many approaches to healthcare delivery are increasingly motivated to prioritize efficiency over relevance, often using methods and strategies borrowed from industry that were never intended to apply to human relationships [18]. In efforts to have clinicians operate at “the top of their licenses,” for example, health systems may prevent clinicians from contextualizing care and biasing them toward intervention (e.g., if all a clinician *can* do is diagnose and treat, that is all he *will* do) [28,29]. Unfortunately, intervention in the absence of understanding can lead to “contextual errors” [30] that increase patient workload and/or limit care effectiveness. Indeed, patients are unlikely to adhere to treatment strategies they do not believe in or seek to achieve clinical targets that are overwhelming, contradictory, or not relevant to them [31].

As such, the MDM Care Model seeks to facilitate legitimate patient partnership. It uses a number of specific strategies and tools to uncover the most appropriate and applicable care for individual, complex patients. It respects the values and preferences of patients and considers ways to acceptably fit health care into the larger purpose of their lives. Informed by new and exciting developments in the fields of psychology and neurobiology, it also introduces innovative and practical methods for leveraging the value of meaningful human relationships (see Box 2).

Box 2 **Traditional chronic disease care for Debbie.**

Three months later, Debbie returns to the clinic with vague complaints of exhaustion and trouble sleeping. Her HgbA1c is 8.9% and her blood pressure is elevated on exam. Upon questioning, it becomes clear that Debbie did not attend the diabetes class. She states that she did not feel comfortable using insulin, and continued taking the pills. Debbie’s clinician reiterates the need for Debbie to use her insulin to improve her diabetes and suggests that attending the diabetes class will give her the confidence she needs to use the insulin and that this will improve her fatigue. Debbie promises to schedule the appointment.

## 4. Strategy 2: Make the Right Care Happen

### 4.1. Prioritize Feasibility

Simply identifying the right care—care that is needed and wanted by patients and feasible for them to enact—is not enough. Practically speaking, this care must actually be carried through. This is a key challenge to efforts aimed at improving the patient-centeredness of care [32]. As such, the MDM Care Model was designed, from the outset, in partnership with implementation scientists who could account for the gamut of factors that impact care effectiveness. Key among these is the resource scarcity that routine settings encounter when attempting to overhaul clinical practice. Thus, the MDM Care Model is designed to permit flexible implementation and its core functions can be carried out in even the most immature care environments.

Specifically, the MDM Care Model is novel in the way it seeks to leverage the value of the clinical encounter in partnering with patients and identifying the most appropriate care strategy. Most models of care delivery, including the Chronic Care Model [33], focus on developing and using infrastructures to coordinate and carry out care activities *presumed* to be applicable. This approach is challenging to implement and may be inadequate [18,34]. Conceptually, the MDM Care Model differs in that it focuses first on identifying truly useful and needed care within the clinical encounter. It then prioritizes the development and maintenance of only those infrastructures essential for carrying relevant care through. In its prioritization of feasibility, MDM posits that making some of the right care happen is better than none at all. In theory, this also facilitates implementation.

### 4.2. Make Sense of It All

MDM attempts to account for the fact that all forms of healthcare delivery require the collective action of people. Traditional models of chronic care delivery often provide recommendations of what could or should be done, generally speaking in broad terms about vaguely defined constructs. These models often have unclear rationales for why certain activities should have value, and provide little guidance on how these activities should be conducted or prioritized [35]. Rarely do they consider the practical realities of how clinicians, teams, and systems will work together to enact the effort.

This is important because, for innovation to take hold and be reliably implemented, it must make sense to those involved [10]. Indeed, without an underlying philosophy to direct care and a shared goal to pursue, care is likely to be less coordinated, less useful, and less effective [10,36,37,38]. Thus, the MDM Care Model is designed to be coherent. It applies the Cumulative Complexity Model to explicitly define a consistent strategy for care optimization and elevates patient goals as the common aim for all care activities. This, in theory, facilitates implementation. The Model also proposes the use of specific interventions to ensure that team members find value, purpose, and satisfaction in the work that they do—a practical matter that impacts organizational productivity and effectiveness [39,40,41]—and that leaders are able to act flexibly and with wisdom to remove barriers to effective care [42] and achieve goals [43]. Similarly, the MDM Care Model seeks to organize care around relationships; this approach is more responsive to the human factors that influence care effectiveness and has shown value in the setting of chronic disease [44,45,46].

### 4.3. Use Available Resources

When MDM is practiced within clinical encounters (*i.e.*, assessments of patient capacity, workload, and complexity occur), it becomes relatively easy to identify the capacity-enhancing resources that patients with multiple chronic conditions and caregivers require to achieve their goals. Many times these resources exist within the healthcare system (as is the case in many integrated care environments), but often times they do not. Indeed, valuable, capacity-enhancing resources exist in almost all communities, but they may be disguised in the form of public health initiatives, social organizations or clubs, community education courses, or faith-based charities, for example. It is well recognized that connections to community resources improve outcomes in the setting of chronic disease [47,48], but these connections are not commonly pursued or achieved [33].

The MDM Care Model makes practical use of the (often freely available) resources that can support patients in their efforts to access care, use care, and enact self-care. This requires the creation and use of resource registries and protocol-based connections. Importantly, the resources included in these registries should be based on legitimate care relationships and explicit, bidirectional understanding of which patients can benefit from (*i.e.*, access and effectively use) which resources and under what circumstances. The Stanford Chronic Disease Self-Management Program, for example, is known to improve patient-centered and disease outcomes and reduce costs [49] and it has been recommended for patients with multiple chronic conditions [3]. Because this program is funded through the Administration on Aging, it is a community-based resource that patients can access free of charge in many areas of the United States.

### 4.4. Monitor and Respond

The natural trajectory of chronic disease is one of gradual yet progressive impact on health and function overlaid with periodic and acute decompensations. This, particularly in its unpredictability, mirrors the trajectory of life. MDM, as already outlined, intends to flexibly account for the complex ways in which health and life interact. It does this by paying careful attention to the dynamic factors that influence patient workload and capacity. At a minimum, this occurs through structured activities within the clinical encounter. In optimal implementation, however, patient-reported measures of context, function, goals, and needs are obtained over time and between encounters. Many methods for obtaining this critical information are likely reasonable and, although specific measures of patient capacity and treatment burden [50] are under development, many suitable and validated measures of related concepts can be helpful. Patient-reported outcomes give practitioners of MDM insight into the life that patients experience beyond the clinic walls. In theory, this aids clinicians in their ability to identify and characterize the workload-capacity balance and to respond appropriately. For example, patients who are recently discharged from the hospital are often in a state of dramatic and acutely low capacity [51]; such patients may be incapable of enacting significant self-management activities and thus may require comprehensive support [24]. On the other hand, when patient capacity is chronically low but reasonably stable and supported, it may be beneficial to encourage patients’ efforts to self-manage their health, develop resilience, and pursue their goals with independence [52]. Deep understanding of the patient experience is needed to make these determinations (see Box 3).

## 5. Minimally Disruptive Medicine: Comprehensive Tools for Implementation

Ultimately, the MDM Care Model is designed to be pragmatically comprehensive, meaning that it seeks to address any and all factors that appear to impact the implementation of effective care for patients with multiple chronic conditions. Most care models focus primarily on optimizing and organizing the system of care delivery or on improving the approach to clinical reasoning. The MDM Care Model aims to do both coherently and together. Indeed, the strategies to achieve MDM (*i.e.*, identifying the right care and making it happen) are operationalized through specific interventions aimed at patients, clinicians, care teams, health system leaders, and community partners among others because all of these stakeholders matter. Similarly, the MDM Care Model makes special efforts to embrace and leverage some of the psychobehavioral and social factors that interact between and among these players; again, because they seem to matter [34,41,53,54].

Box 3 **The switch of Debbie’s care to MDM.**

Soon after Debbie’s appointment, the clinic decides to implement the MDM Care Model for patients with multiple chronic conditions. Clinicians go through a 1-hour training program and begin using workload and capacity screening tools within the encounter. Clinic leaders also begin holding bi-weekly team huddles to address relationship and communication problems identified in staff surveys. Debbie is invited to attend one of these huddles to help the care team make sense of their efforts. At the huddle, Debbie agrees to re-enact her previous encounter according to the MDM Model. Using a capacity assessment tool, the team uncovers that Debbie’s capacity had been constrained by physical limitations and problems with her emotions. Debbie states that she had been doing well until she sprained her ankle. Because she was unable to stand or exercise, Debbie had taken off work and was spending much of her time lying on her couch. At the same time, Debbie’s son was getting into trouble at school and she was concerned about the unfavorable influence of some of his friends. During a workload assessment, Debbie remarks that she had intended to do a better job of taking care of her health, but that she was beginning to feel overwhelmed and sad. Although she wanted to attend the diabetes class and start using insulin, she was just getting back to work and the program was only offered in the mornings. Debbie gets off work at 3 p.m.

A comprehensive manual for implementation of the MDM Care Model is currently in preparation and will soon be available to patients, clinicians, researchers, health systems, and policy-makers. A summary of some of the tools that can be used are outlined in Table 1. All tools map, conceptually, to the Cumulative Complexity Model. Some focus on identifying the right care for patients with multiple chronic conditions. These help to direct and facilitate the implementation of other tools and strategies aimed at reducing patient workload and/or enhancing patient capacity.

## 6. Conclusions

### 6.1. The MDM Difference

To date, efforts to address the unique challenges of caring for patients with multiple chronic conditions have had mixed results [55]. An innovative approach to caring for these patients is urgently needed [56]. The MDM Care Model is novel in that it considers and incorporates two factors that are particularly relevant to patients with multiple chronic conditions: treatment burden [57] and complexity. Indeed, the MDM Care Model comprises a toolkit of interventions that map to a conceptual framework—the Cumulative Complexity Model. Importantly, this framework includes treatment burden as a core construct and may also manifest expressions of underlying theories of complexity (*i.e.*, emergent syndromes of workload-capacity imbalances). Although several patient-centered models of healthcare delivery exist [58,59,60], rarely are these oriented to explicitly care for patients with multiple chronic conditions. Those that are [56,61,62,63,64,65], often promote patient-centered ideas or concepts without providing specific instruction on how to implement them. Rarely do they link to a testable framework. These issues may limit the ability of these models to build an evidence base for the value of holistic and generalist-oriented approaches to caring for patients with multiple chronic conditions [56]. The MDM Care Model describes a theoretical relationship between testable constructs that affect care. This permits further validation and refinement of the Model and the rational development of interventions specific to it (see Box 4).

**Table 1:** The minimally disruptive medicine (MDM) toolkit.

Tool	Description
**Tools to Identify the Right Care**	
Goal-elicitation	An attempt to identify transcendent patient goals for life that can be entered into the medical record and used to orient care
Patient partnerships	A structured commitment among patients and clinicians to work together to identify the right care and to make the right care happen
Shared decision making	Used to incorporate patient values and preferences into management decisions, legitimize partnership, and arrive at feasible care strategies
Capacity assessments	Structured, within-encounter screens used to identify contextual limitations in patient capacity that impact care effectiveness and that may be amenable to support or intervention
Workload assessments	Structured, within-encounter screens used to identify the intrusiveness of health on life and to find opportunities for treatment plan augmentation
Capacity coaching	Between-clinician encounter interactions that screen for progress in goals and the influence of capacity and workload on health and wellness
Patient-reported outcome tracking	Systematic, ongoing recording of patient-reported health status, burdens of life and health, and changes in the quality or availability of support
**Tools to Make the Right Care Happen**	
Resource registries	Lists of resources within and outside of the health system that have explicit and predefined agreements to provide specific support to specific patients
Lean consumption	Healthcare provider-initiated efforts to improve the efficiency of interacting with health care from the patient’s perspective (e.g., by shortening waiting times, streamlining administrative hurdles)
Medication therapy management	A version of medication therapy management that focuses on optimization of medication regimens in regards to not only patient need, but also want and fit; best implemented with the power to “deprescribe” low-value and burdensome medications
Community navigators	Individuals that can be used to more intimately connect patients to community resources
Relational coordination	Method of organizing care based on shared aims and understanding that uses specific analytics to identify within-team relationships that are impeding care effectiveness
Wisdom leadership	Training for team and system leaders to enhance their capacity to lead with wisdom and to remove barriers impeding care effectiveness—often through the use of reflective team huddles
Choosing Wisely Campaign	Used to promote a practice or system-level culture of parsimonious and patient-centered care and to establish social norms that counteract the bias toward intervention

Box 4 **Sustained, minimally disruptive care for Debbie.**

Upon understanding the complex ways in which Debbie’s capacity, workload, and context impacted her care, the MDM Care Team was energized to implement more of the Model and to tailor Debbie’s management approach. Specifically, the diabetes education classes were rescheduled to offer morning and late afternoon options; an online version was also planned. Debbie and her clinician had a discussion about Debbie’s preferences for diabetes management and they decided together that, for now, Debbie would focus on optimizing her mood, being there for her son, and getting back to work. Debbie was invited to partner with the care team to start building a resource registry. The first resource added was a community art interest group. Debbie attends every week and in this she is finding fellowship and respite from her stressful life. Although she is yet to start insulin, her HbA1c is now 7.4%. She is feeling better.

### 6.2. Opportunities for Further Study

Although the MDM Care Model has theoretical value in optimizing care for patients with multiple chronic conditions, it remains incompletely tested. Indeed, the objectives of this paper are to outline the theoretical rationale for the Model and to propose some practical care strategies that are consistent with it. Although many of the toolkit components have shown value when tested in isolation, it remains unknown if they have synergistic value when oriented by a conceptual framework. Future study should aim to test the MDM Care Model more completely. This will likely require the development and validation of measures of key constructs in the Cumulative Complexity Model (*i.e.*, treatment burden and capacity) and more complete understanding of the practical identification and treatment of workload-capacity imbalances. Additional study will be needed to assess the barriers and facilitators to implementation of MDM interventions and, specifically, if orientation by the theoretical framework affects this process. Such efforts may have value in identifying an effective means of caring for patients with multiple chronic conditions.
